# Comparison of two iron supplementation methods on Hemoglobin level and Menstrual Bleeding in Tabriz students

**Published:** 2014-03-15

**Authors:** S Bani, A Hassanpour- Siahestalkhi, Sh Hassanpour, S Mommad- Alizadeh- Charandabi, M Mirghafourvand, Y Javadzadeh

**Affiliations:** 1 Instructor, Department of Midwifery, Tabriz University of Medical Sciences, Tabriz, Iran; 2 Student in Midwifery, Tabriz University of Medical Sciences, Tabriz, Iran.; 3 Assistant Professor, Department of Midwifery, Tabriz University of Medical Sciences, Tabriz, Iran; 4 Associate Professor of Pharmaceutical Technology, Faculty of Pharmacy Tabriz University of Medical Sciences, Tabriz, Iran.

**Keywords:** Hemoglobin, Iron supplement, Menstrual bleeding

## Abstract

**Background:**

Iron deficiency anemia is a global health problem, and approximately 50% of anemia is caused by iron deficiency. According to studies, iron supplementation in young females improves iron status by increasing concentration of hemoglobin. To compare of prescribing two methods of iron supplementation administered either on a weekly basis or during menstruation, on hemoglobin level and menstrual blood¸ this double blind Randomized clinical trial study was carried out among female students in Tabriz, Iran.

**Materials and Methods:**

In this double-blind randomized clinical trial, 150 female students allocated randomly in two groups. (75 students took an iron tablet weekly and 75 students took an iron tablet for first four days during their menstruation cycle for 16 weeks). Before and after intervention, the level of hemoglobin was measured and Higham chart was completed by participants in each group. Chi-square, independent t-Test, paired t-Test and ANCOVA were used for data analysis.

**Results:**

There was no significant difference between two groups in terms of demographic characteristics, hemoglobin level and amount of menstrual bleeding before and after intervention (p>0.05). Taking iron supplement increased significantly the level of hemoglobin in each group (p<0.001). However, there was no significant difference in amount of menstrual bleeding (p>0.05) when comparing the data before and after intervention in each group.

**Conclusion:**

The two iron supplementation methods (menstrual bleeding period and weekly) have similar results on Hemoglobin level and menstrual bleeding.

## Introduction

Iron deficiency anemia (IDA) is a global health problem, and is considered as the most common chronic disease (1). Although all the individuals can be affected by this problem, the highest incidence is in reproductive- age women (2, 3). According to WHO, the prevalence of anemia in women ages between 15 to 49 is 33.4% (4). In another study, the prevalence of iron deficiency in reproductive age girls in East Azerbaijan was 13% (5). Anemia is caused by a variety of reason, but almost 50% of anemia is due to iron deficiency (6). Iron deficiency anemia (IDA) is a nutritional deficiency disease that could lead to a maternal and neonatal mortality. (7, 8).

Menorrhagia (unusual menstrual bleeding) occurs in all age groups of women, but more than half of the women with menorrhagia are under 40 years. Menorrhagia is one of the most common reasons of IDA in women (9-12). Hallberg and Rossander (1991) in a study entitled "Iron requirements in menstruating women" showed that iron deficiency increases by menstrual bleeding and at the same time level of hemoglobin decreases (13). But in a study by Clancy et al. (2006), menstrual bleeding was not considered a risk factor for IDA (14).

It is possible to cure anemia and reduce its consequences in early stage by doing required tests for diagnosing women's anemia and prescribing appropriate supplements and nutritional educations. Taking appropriate supplements in lifetime is the simplest and cheapest way to prevent from chronic diseases.

Although WHO has recommended taking iron pills once in a week for 12 weeks and Iron supplementation Committee has recommended it once in a week for 16 weeks for all the women of childbearing age (15 to 49) (15).Various researchers in Iran especially Khademlouet al. (2006) have compared the effect of taking iron supplement daily and weekly on the level of hemoglobin and showed that taking weekly iron supplement has the same effect as taking it daily (16). However, according to the investigations, no study has been conducted to compare the effect of iron supplements during menstruation period and weekly on anemia and amount of menstruation bleeding in Iran and it seems taking iron supplement in menstrual bleeding period increases individual's motivation for taking this pills, the aim of this study is comparison of the effect of iron supplement prescription with two methods of weekly and in menstrual bleeding period on hemoglobin level and amount of menstrual bleeding in students of Tabriz high schools.

## Materials and Methods

This research was a double-blind randomized clinical trial that was conducted from January until May 2013. The study population was female students of Tabriz¸ and the period of intervention for each participant was 4 months. By using STATA (version 9.2) and according to the study by McClung et al. (2009) (17) and by considering the least difference of 5 percent between the groups (M1=11.6, SD1=1.3, SD2=1.2, M2=12.2, level of significance=0.05, and statistical power=80%), the number of samples were estimated 69 individuals for each group. By considering the probability of attrition, it was estimated 75 individuals for each group.

The eligibility criteria of this study were: anemia based on clinical examination (such as examining conjunctiva, mucosal discoloration, nails etc, not taking vitamin and iron supplement in last 3 months, not taking oral or intravenous steroid hormonal drugs, not having liver diseases, infectious diseases and parasitic diseases, not having blood diseases such as Thalassemia and Hemophilia, not having medical prohibition for taking iron, not having mental illness, not having systemic disease such as diabetes, thyroid problems and overactive adrenal glands according to the individual herself.

After getting permission from the Ethics Committee of Tabriz University of Medical Sciences (code: 91111), the volunteer students who had all the criteria, were invited to participate in this study. Informed written consent was obtained from participants. Then 2.5cc of total blood samples were taken and then poured into the vials containing anticoagulant (EDETA) and shacked 2 to 3 times to prevent clotting, and then were transferred to the laboratory. In the laboratory, the vials were placed on shaker for 5 to 10 minutes to be mixed with anticoagulants. Then they were placed under the probe of Cell Counter Device (model DC-3000 the mindry company– made in china) to calculate the amount of hemoglobin. If the hemoglobin level were less than 12 mg/dl, they could enter into the study. 

This trend was continued up to 150 individuals (75 individuals in each group). The students were divided into two groups of weekly (A) and menstrual iron supplement (B) using block randomization with block sizes of 4 and 6 and allocation ratio of 1:1. In order to allocation concealment, the tablets were placed in closed envelopes and the envelopes were numbered respectively. Researcher and participants were unaware from type of supplementations (Double Blinding), and another person put the drugs in uniform envelopes with A and B cods and then gave them to researchers.

Before prescribing iron supplement, questionnaire of demographic and menstrual characteristics and also higham chart were completed by participants. Higham is a standard visual scale for assessing the amount of bleeding (18, 19). To increase the accuracy of estimated blood loss during menstruation, sanitary pads produced by a specific manufacturer (The Nava Behdasht Company in Iran) were given to the participants.

Content validity was used for the assessing validity of the questionnaire of demographic and menstrual characteristics. Reliability was determined by Spearman's correlation coefficient that was 0.9.

The pills contained 60mg elemental iron and 400 micro-gram folic acid. Raw materials of iron supplements (ferrous sulfate and folic acid) were produced by the company of Daroupakhsh in Tehran and were prepared and packaged by laboratory of pharmacy school, Tabriz University of Medical Sciences. The length of intervention was 16 weeks.

In weekly iron supplement, the iron pills were taken on a certain day of a week and in menstrual iron supplement, the iron pills were taken on first 4 days of menstrual cycle. In order to control the parasitic infection, a single dose of Mebendazole (500mg) was prescribed to all students for three days before the iron supplementation. One week after the last iron pill prescription in each group, the level of hemoglobin was measured again. Also after the end of intervention period, higham chart was completed by the participants. 

**Statistical Analysis**

The data was analyzed using SPSS/ver 13 software. Chi-square, independent t-Test, paired t-Test and ANCOVA were used.

## Results

**Figure1:**
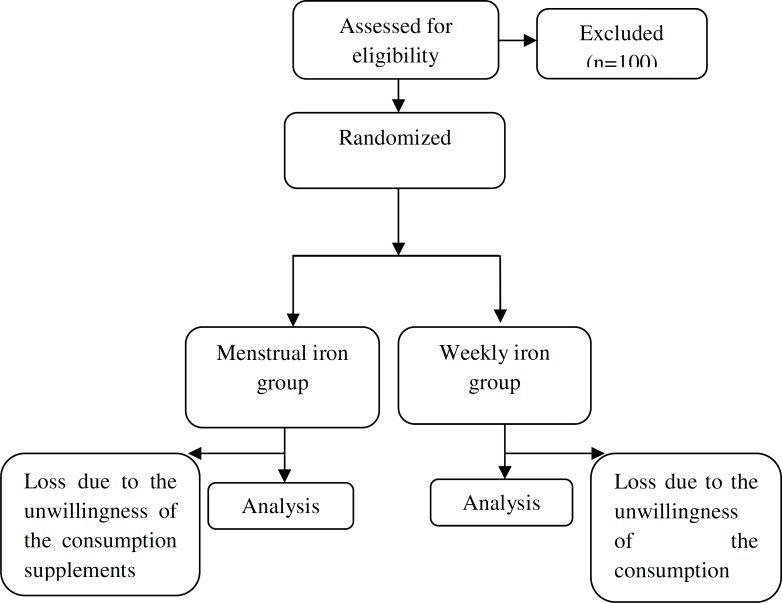
Flowchart of participants in the study

150 individuals participated in this research (75 individuals in each group) that 9 individuals of first group (weekly iron) and 6 individuals of second group (menstrual iron) left the research because of unwillingness to continue iron supplements (Figure1). There was no significant difference in demographic characteristics and menstrual history between two groups (Table 1). There was no significant difference between two groups in level of hemoglobin before the intervention (p>0.05). 

Also after iron supplementation,there was no significant difference in level of hemoglobin by controlling the hemoglobin level before intervention between two groups (p>0.05). 

By comparing before and after iron supplementation in each group, there was a significant difference in amount of hemoglobin (p<0.001) as taking iron supplement increased the amount of hemoglobin (Table 2). There was no significant difference between two groups considering the amount of menstrual bleeding before iron supplementation (p>0.05). Also after intervention there was no significant difference in amount of menstrual bleeding by controlling the amount of menstrual bleeding before intervention between two groups (p>0.05). Also by comparing after and before intervention in each group, there was no significant difference in higham score (p>0.05) (Table 2). There were no side-effects in participants as a result of taking supplement.

**Table I:** Demographic – Social of characteristics the subjects divided to groups weekly iron and menstrual iron

**Results**	**Menstrual iron group** **69** **=** **N**	**Weekly iron group** **66** **=** **N**	**Characteristic**
**P=0.453** **†**	(34%) 23 (61%) 42 (6%) 4	26(40%) 37(56%) (5%) 3	**Age (years)** 15> 17-15 17<
**P=0.395** **‡**	*(2.1) 21.2	*(2.2) 20.9	**Body Mass Index** ^§^ ** (Kg/m** ^2^ **)**
**P=0.928** **†**	(51%)35 (45%)31 (5%)3	(52%)34 (43%) 28 (6%) 4(6	**Menarche age** 13> 15-13 15<
**P=0.583** **§**	(68%) 47 (32%) 22	(64%) 42 (37%) 24	**Menstrual cycle** Regular Irregular
**P=0.886** **†**	(86%) 59 (15%) 10	(87%) 57 (14%) 9	**Duration of menstrual bleeding** 7≥ 7<
**P=0.064** **†**	(3%) 2 (73%) 50 (25%) 17	(9%)6 (76%) 5 (15%) 1	**Severity of menstrual bleeding** **¥** Mild Moderate Severe

All numbers except Resources of Specified, to form of frequency (percent) is shown. ‡Independent t-tests, * Mean (SD), ¥The self-reported, †Chi-square test for trend, §Chi-square test, § Body Mass Index (BMI) by dividing weight in kilograms to square of height in meters were obtained

**Table II:** Comparison of hemoglobin level and menstrual bleeding in two groups weekly and menstrual iron

**p**	**Menstrual iron group** **N=69**	**Weekly iron group** **N=66**	**Outcome**
	^*^ ** Mean (SD)**	* ** Mean (SD)**	
			**Hemoglobin level**
**0.062** **†**	(0.5)11.1	(0.4)11.3	**Before intervention**
**0.727** **‡**	(0.9)11.8	(0.8)11.9	**After intervention**
	(1.1)0.7	(0.9) 0.6	**Difference before and after**
	0.001>	0.001>	**P** ^§^
			**Higham score**
**0.437** **†**	(38.3)56.2	(30.7) 51.5	**Before intervention**
**0.725** **‡**	(37.2)54.6	(36)53.8	**After intervention**
	(33.4) 1.5-	(34.5)2.2	**Difference before and after**
	0.696	0.595	**P** ^§^

*Mean (SD),†Independent T-Tests,‡ Analysis of covariance(ANCOVA),^§^Paired Sample T-Test

## Discussion

The risk of IDA in women of childbearing age is very considerable because of additional needed iron related to menses and pregnancy (20). According to the results of this study, taking iron supplement both weekly and in menstrual bleeding period will result in increasing the amount of hemoglobin. according to a study by Februhartanty et al. (1998) in Indonesia, taking iron supplement both weekly and during menstrual period in female students increased the concentration of hemoglobin (21). In study by Tee et al. (1997) in Malaysia, the weekly use of iron – folate increased the mean of hemoglobin consistently and significantly in relation to the duration of treatment (22). According to the study by McClung et al. (1997) that was conducted on female soldiers during educational period in US, iron supplement could improve hemoglobin state (17). The results of present study correspond with the results of the mentioned studies. 

In this research, there was no significant difference in amount of hemoglobin between two methods of prescribing iron supplement (weekly and during menstrual bleeding), but both methods improved hemoglobin levels; while the results of study by Februhartanty et al. (1998) showed that weekly iron supplement is preferable (21). According to the study by Schultink (1998) in Indonesia, accepting weekly iron supplement was less probable (23). On the other hand, losing iron in menstruation is the principal source of change in iron requirements of non-pregnant women and in menstrual bleeding period (12, 24). The difference between the result of our research and the result of study by Februhartanty et al. (1998) may be because of geographical changes and life style such as certain nutritional patterns. This research presented similar results in taking two methods of prescribing iron supplement and showed that two methods of prescribing iron supplement have the same effects. Because the menstrual bleeding period is an exclusive period due to blood loss¸ it seems more likely to take the supplements in this period, so it can be considered as a substitute method.

In this study, there was no significant difference after the intervention between two groups regarding the amount of menstrual bleeding. In a study by Taymor et al. (1964), in 74 of 83 patients suffering from menorrhagia and with lower iron level and without any provable pathologic disease, there was a satisfactory respond to iron treatment and menorrhagia was improved. The patients, who did not respond to treatment, had a high rate of organic pathology. The mentioned study showed that chronic iron deficiency and anemia can be a cause as well as a result of menorrhagia. (25). The difference between the results of that study and our study may be related to iron dose and its duration of prescription. Also, in our study, the participants' higham scores were less than 100, while the mentioned study was conducted on women suffering from menorrhagia.

This study showed that the result of iron supplementation prescription both weekly and in menstrual bleeding period, is the same on level of hemoglobin and amount of menstrual bleeding.

 It is suggested that more studies with more students and more iron dose should be conducted on other childbearing ages in different regions of Iran and on the persons suffering from menorrhagia (with higham score more than 100).

## Conclusion

The two iron supplementation methods (menstrual bleeding period and weekly) have similar results on Hemoglobin level and menstrual bleeding. Since, it seems iron supplementation in menstrual bleeding period is more acceptable, can be replaced for weekly iron supplementation.

## References

[1] DeMaeyer E, Adiels-Tegman M (1985). The prevalence of anaemia in the world. World Health Stat Q.

[2] Dallman PR, Yip R, Johnson C (1984 Mar). Prevalence and causes of anemia in the United States, 1976 to 1980. Am J Clin Nutr.

[3] Kuvibidila S, Yu L, Warrier RP, Ode D, Mbele V (1994 Jun). Usefulness of serum ferritin levels in the assessment of iron status in non-pregnant Zairean women of childbearing age. J Trop Med Hyg.

[4] WHO (2007). WHO global database on anaemia ( Islamic Republic of Iran ). http://who.int/vmnis/anaemia/data/database/countries/irn_ida.pdf.

[5] Pourghassem B, Kimiagar GSM, Abolfathi AA, Vallaii N, Ghaffarpour M (2000). Prevalence of iron deficiency, anaemia, and iron-deficiency anaemia in high-school students in Jolfa, East Azerbaijan. Food Nutr Bull.

[6] WHO (2001). Iron deficiency anemia: assessment, prevention, and control. A guide for programme managers. http://www.who.int/nutrition/publications/micronutrients/anaemia_iron_deficiency/WHO_NHD_01.3/en/.

[7] Macgregor M (1963). Maternal anemia as a factor in prematurity and prenatal mortality. Scott Med J.

[8] Scholl TO, Hediger ML (1994 Feb). Anemia and iron-deficiency anemia: compilation of data on pregnancy outcome. Am J Clin Nutr.

[9] Claessens EA, Cowell CA (1981). Acute adolescent menorrhagia New York: Mac graw- Hill. Am J Obstet Gynecol.

[10] Hallberg L, Hogdahl AM, Nilsson L, Rybo G (1966). Menstrual blood loss-a population study. Variation at different ages and attempts to define normality. Acta Obstet Gynecol Scand.

[11] Hurskainen R, Teperi J, Paavonen J, Cacciatore B (1999). Menorrhagia and uterine artery blood flow. Hum Reprod.

[12] Jacobs A, Butler EB (1965). Menstrual blood loss in iron-deficiency anemia. Lancet.

[13] Hallberg L, Rossander-Hulten L (1991). Iron requirements in menstruating women. Am J Clin Nutr.

[14] Clancy KBH, Nenko I, Jasienska G (2006). Menstruation Does Not Cause Anemia: Endometrial Thickness Correlates Positively with Erythrocyte Count and Hemoglobin Concentration in Premenopausal Women. Am J Hum Biol.

[15] Scientific Committee of iron supplementation, Office of improves the Community Nutrition Department of Health, Health Center of East Azarbaijan (2007). Program to iron supplementation of groups at risk of iron deficiency anemia.

[16] Khademlou M, Ajami A, Khalilian A, Moatamed N (2006). Effect of administration of weekly and daily iron supplementation on hemoglobin and serum ferritin levels in pregnant women referring to health centers - Care rural city of Sari. Journal - Mazandaran University of Medical Sciences.

[17] McClung JP, Karl JP, Cable SJ, Williams KW, Nindl BC, Young AJ (2009). Randomized, double-blind, placebo-controlled trial of iron supplementation in female soldiers during military training: effects on iron status, physical performance, and mood. Am J Clin Nutr.

[18] Biri A, Bozkurt N, Korucuoglu U, Yilmaz E, Tiras B, Guner H (2008). Use of pictorial chart for managing menorrhagia among Turkish women. Turkish- German Gynecol Assoc.

[19] Higham JM, O'Brien PM, Shaw RW (1990). Assessment of menstrual blood loss using a pictorial chart. Br J Obstet Gynaecol.

[20] Hallberg L (1995). Results of surveys to assess iron status in Europe. Nutr Rev.

[21] Februhartanty J, Dillon D, Khusun H (2002). Will iron supplementation given during menstruation improve iron status better than weekly supplementation. Asia Pac J Clin Nutr.

[22] Tee ES, Kandiah M, Awin N, Chong SM, Satgunasingam N, Kamarudin L (1999). School-administered weekly iron-folate supplements improve hemoglobin and ferritin concentrations in Malaysian adolescent girls. Am J Clin Nutr.

[23] Schultink W (1996). Iron-supplementation programmes: Compliance of target groups and frequency of tablet intake. Food Nutr Bull.

[24] Hallberg L, Hogdahl AM, Nilsson L, Rybo G (1966). Menstrual blood loss and iron deficiency. Acta Med Scand.

[25] Taymor ML, Sturgis SH, Yahia C (1964). The Etiological Role of Chronic Iron Deficiency in Production of Menorrhagia. JAMA.

